# Persistence of Neutralizing Antibody Against Dengue Virus 2 After 70 Years from Infection in Nagasaki

**DOI:** 10.1089/biores.2016.0016

**Published:** 2016-07-01

**Authors:** Mya Myat Ngwe Tun, Yoshihito Muta, Shingo Inoue, Kouichi Morita

**Affiliations:** ^1^ Department of Virology, Institute of Tropical Medicine, Leading Graduate School Program, Nagasaki University, Nagasaki, Japan.; ^2^ Muta Hospital, Omura City, Japan.

**Keywords:** dengue, Japan, neutralization antibody

## Abstract

This study aimed to investigate the duration of humoral immune responses to dengue virus (DENV) infection in Japanese who experienced acute febrile illness with hemorrhagic manifestations 70 years ago, when an epidemic of dengue occurred in Nagasaki, Japan, from 1942 to 1944. A Japanese volunteer requested serological diagnosis of DENV infection in 2014 and donated blood sample to measure the antibody titer against DENV by antiflavi IgG indirect ELISA, focus reduction neutralization test, and plaque reduction neutralization test. The serum sample of the volunteer was positive in flavi IgG ELISA and it indicated primary infection. In the neutralization test, the highest neutralizing titer was ≥218 for DENV-2. We report here the existence of DENV-specific antibodies in the serum of a person after 70 years from infection. Published reports indicated that DENV-1 was responsible for the 1942–1944 outbreak in Nagasaki. However, our data suggested that DENV-2 also played a role in this Nagasaki dengue epidemic.

## Introduction

Dengue virus (DENV) is a member of the genus *Flavivirus*, family *Flaviviridae*. DENV is transmitted by infected mosquitoes, mainly *Aedes aegypti* and *Aedes albopictus*. The four serotypes of DENV (DENV-1 to DENV-4) are antigenically and genetically distinct. Infection by any DENV serotype can cause mild dengue fever (DF), severe dengue hemorrhagic fever, or the more severe dengue shock syndrome, as described in the 1997 World Health Organization classification system of the disease severity. This is now categorized into dengue with or without warning signs and severe dengue.^1^

DENV infection is found in tropical and subtropical regions around the world such as Southeast and South Asia, Pacific, Central and South America, the Caribbean, Africa, and Europe. In Japan, dengue epidemics were recorded in the Main Islands (1942–1945) once before the end of World War II and several times (1893–1955) in the Okinawa Islands.^2,3^ A dengue outbreak due to DENV-1 occurred in Osaka, Kobe, Hiroshima, and Nagasaki from 1942 to 1945.^4^ In Okinawa, the outbreak was due to DENV-1 and DENV-2.^5^ After several decades without confirmed autochthonous cases of DF in Japan, a patient with no history of overseas travel was reported to contract DF in Tokyo, and as of October 2014, a total of 160 autochthonous cases were confirmed in this country.^6^ This study aimed to investigate the duration of humoral immune responses to DENV infection in a Japanese person who experienced acute febrile illness with hemorrhagic manifestations 70 years ago, when an epidemic of dengue occurred in Nagasaki, Japan, from 1942 to 1944.

## Materials and Methods

### Viruses and cell lines

The viruses used for anti-flavi IgG ELISA, focus reduction neutralization test (FRNT_50_), and plaque reduction neutralization test (PRNT_50_) were as follows: DENV-1 (strain: M-120), DENV-2 (strain: M-58), DENV-3 (strain: SLMC50), DENV-4 (strain: SLMC318), and Japanese encephalitis virus (strain: JaOArS982). These viruses were propagated in the C6/36 mosquito cell line and were used to inoculate the Vero and baby hamster kidney (BHK) cell lines for virus titration and neutralization tests.

### Detection of flavi IgG

In-house flavi IgG indirect ELISA was carried out following the protocol described previously.^7,8^ The plate was coated with purified Japanese encephalitis virus (JEV) antigen (250 ng/100 μL/well) and incubated at 4°C overnight. Wells were blocked with Block Ace and were incubated at room temperature (RT) for 1 h. After incubation, wells were washed with phosphate-buffered saline (PBS) containing 0.05% Tween 20 (PBS-T) three times. Test samples and positive and negative controls were diluted 1:1000 in PBS-T and 100 μL aliquots were distributed into duplicate wells. The plate was incubated at 37°C for 1 h and then washed as already described. After washing, 1:30,000 dilution of HRP-conjugated antihuman IgG (American Qualex) was added at 100 μL/well and plate incubated at 37°C for 1 h. The plate was washed and *o*-phenylenediamine dihydrochloride substrate at 100 μL/well was added and kept in the dark at RT for 1 h. To terminate the reaction, 100 μL of 1 N sulfuric acid was added to each well.

The optical density (OD) was read at 492 nm (Multiscan JX). A standard curve was prepared using the OD values of the dengue-positive control serum samples starting with a 1000-fold dilution, followed by serial 2-fold dilutions. A sample titer equal to or greater than 1:3000 was considered to be positive. DENV primary and secondary infections were determined at a sample titer <52,000 and ≥52,000, respectively.

### Focus reduction neutralization test

To confirm neutralizing activity of DENV and JEV-specific antibodies, neutralization tests were done.^9^ Patient serum samples were heat inactivated at 56°C for 30 min before the assay. Serum samples were serially diluted twofold with 2% fetal calf serum (FCS) in Eagle's minimum essential medium (MEM). A volume of 150 μL of each dilution of the serum samples was mixed with an equal volume of DENV or JEV, which contained 60 focus-forming units. This was followed by incubation at 37°C for 1 h for a virus–antibody neutralization reaction. The virus and serum mixture was inoculated onto Vero cell monolayer in a 96-well plate at 37°C for 1 h. After incubation, the infected cells were overlaid with 1.25% methylcellulose 4000 in 2% FCS in MEM. The plates with JEV and with DENV were then incubated at 37°C for 36 h and 3 days, respectively. The plates were washed with PBS, fixed with 4% paraformaldehyde phosphate buffer solution for 30 min at RT, rinsed, and cells in each well were permeabilized with 1% NP-40 solution in PBS for 30 min at RT. After washing, the plates were blocked with Block Ace for 30 min at RT. A pool of human serum samples with a high titer for antiflavivirus IgG (diluted 1:1500) was then added, and plates were incubated at 37°C for 1 h and washed. Subsequently, 1:1500-diluted HRP-conjugated goat antihuman IgG was added to the plates and incubated at 37°C for 1 h. Staining of cells was done by the addition of a 0.5 mg/mL solution of substrate 3,3′-diaminobenzidine tetrahydrochloride in PBS with 0.03% of H_2_O_2_, and the staining reaction was allowed to proceed for 10 min at RT. After washing the stained cells, the number of foci of stained cells per well was counted under a microscope.

The reciprocal of the endpoint serum dilution that provided a 50% or greater reduction in the mean number of foci relative to the control wells that contained no serum was considered to be the FRNT_50_ titer. On the basis of the previously published criteria of neutralizing antibody patterns,^8,10^ a monotypic antibody response (primary infection) was defined as neutralization titer ≥1:10 to only one serotype, or neutralization titer ≥1:10 for more than one serotype but 1:80 for only one serotype. In monotypic pattern of anti-DENV neutralizing antibodies, the DENV serotype with the highest FRNT_50_ was assumed to be the infecting serotype. Multitypic antibody response (secondary infection) was defined as neutralization titer >10 for more than one serotype without a neutralization titer ≥80 to only one serotype.

### Plaque reduction neutralization test

Heat-inactivated human serum samples were serially diluted twofold in 2% FCS in MEM. One hundred fifty microliters of each dilution of the serum samples was mixed with an equal volume of virus, which contained 60 plaque-forming units, followed by incubation at 37°C for 1 h for a virus–antibody neutralization reaction. One hundred microliters of each mixture was then used to inoculate BHK cell monolayer in a 24-well plate. After incubation at 37°C for 1 h, the infected cells were overlaid with 500 μL of MEM containing 2% FCS and 1% methylcellulose. The plates were then incubated at 37°C for 3–5 days based on the virus and dengue serotypes. The plate was washed with PBS to remove methylcellulose, fixed with 4% paraformaldehyde phosphate buffer solution, and stained with 0.1% crystal violet in 10% ethanol. The reciprocal of the endpoint serum dilution that provided a 50% or greater reduction in the mean number of plaque relative to the control wells that contained no serum was considered to be the PRNT_50_ titer.

## Results and Discussion

We investigated the duration of humoral responses to DENV infection of an 84-year-old Japanese male, who experienced acute febrile infection with hemorrhagic manifestations when he was a 13-year-old junior high school student in Nagasaki in August 1944. During that period, there was a dengue outbreak in Nagasaki. He was clinically diagnosed to have DF. His classmates and his neighbors experienced clinical symptoms similar to what he had, such as severe headache, fatigue, muscle pain, high fever, severe nasal bleeding, vomiting with blood, and bloody stool. After 70 years from this experience, this person requested serological diagnosis of DENV infection in October 2014 and volunteered to donate his blood sample. The volunteer has no travel history to dengue endemic countries and he resided in a place where there were no autochthonous cases of dengue. He had also not experienced the same symptoms of dengue for the second time. He has a JEV vaccination history.

Table 1 shows the results of ELISA, FRNT_50_, and PRNT_50_ of the serum sample. The serum sample of the volunteer was flavi IgG ELISA positive, and based on the IgG titer, it indicated primary infection. In the neutralization test, the highest neutralizing titer was found in DENV-2. Presence of DENV-1, -3, and -4, and JEV neutralizing antibodies was detected, but their levels were low. Based on the previously published criteria of monotypic pattern of anti-DENV neutralizing antibodies, the DENV serotype with the highest neutralization titer was assumed to be the infecting serotype. Thus, it is most likely that the patient was infected with DENV-2.

**Table 1.** **Neutralization Antibodies Profile of a Japanese Person Who Reported Experiencing Dengue Fever-Like Illness in 1944**

Patient	Age (year)	Sex	Flavi IgG titer	Neutralization test	DENV-1 M-120	DENV-2 M-58	DENV-3 SLMC50	DENV-4 SLMC318	JEV S982
1	83	M	31,083	FRNT_50_	71	218	30	32	55
				PRNT_50_	29	238	20	20	36

DENV-1 M-120, Myanmar strain (M-120/2013); DENV-2 M-58, Myanmar strain (M-58/2013); DENV-3 SLMC 50, Philippine strain (SLMC50); DENV-4 SLMC 318, Philippine strain (SLMC318); FRNT_50_, 50% focus reduction neutralization test; JEV-S982, Japan strain (JaOArS982); PRNT_50_, 50% plaque reduction neutralization test.

In several studies, long-term existence of antiflavivirus antibodies has been reported. Fujita et al.^11^ mentioned that in their study one of the patients who stayed in Nagasaki had DENV-2 neutralizing titer a little higher than DENV-1. The question of whether the DENV-2 was involved in the Nagasaki epidemic deserves further consideration.^11^ Taken together, our data strongly suggest that DENV-2 played a role in the Nagasaki dengue epidemic in 1942–1944. The long-term persistence of antiflavivirus neutralizing antibody up to 30–60 years has been demonstrated in several studies.^12^ We report here the existence of antibody against DENV in the serum of a person who got infected 70 years ago.

## Abbreviations Used

BHK: baby hamster kidney
DENV: dengue virus
DF: dengue fever
FCS: fetal calf serum
FRNT_50_: focus reduction neutralization test
JEV: Japanese encephalitis virus
MEM: Eagle's minimum essential medium
OD: optical density
PBS: phosphate-buffered saline
PRNT_50_: plaque reduction neutralization test
RT: room temperature
